# The behavioral and physiological correlates of affective mood switching in premenstrual dysphoric disorder

**DOI:** 10.3389/fpsyt.2024.1448914

**Published:** 2024-11-04

**Authors:** Robin Dara Brown, Erin Bondy, Julianna Prim, Gabriel Dichter, Crystal Edler Schiller

**Affiliations:** ^1^ Department of Psychology and Neuroscience, University of North Carolina-Chapel Hill, Chapel Hill, NC, United States; ^2^ Department of Psychiatry, University of North Carolina-Chapel Hill, Chapel Hill, NC, United States; ^3^ Carolina Institute for Developmental Disabilities , University of North Carolina at Chapel Hill School of Medicine, Chapel Hill, NC, United States

**Keywords:** PMDD, PMS, remote monitoring, sleep, HRV, physical activity

## Abstract

Premenstrual dysphoric disorder (PMDD), a more severe manifestation of premenstrual syndrome (PMS), is characterized by emotional, behavioral, and physical symptoms that begin in the mid-to-late luteal phase of the menstrual cycle, when estradiol and progesterone levels precipitously decline, and remit after the onset of menses. Remotely monitoring physiologic variables associated with PMDD depression symptoms, such as heart rate variability (HRV), sleep, and physical activity, holds promise for developing an affective state prediction model. Switching into and out of depressive states is associated with an increased risk of suicide, and therefore, monitoring periods of affective switching may help mitigate risk. Management of other chronic health conditions, including cardiovascular disease and diabetes, has benefited from remote digital monitoring paradigms that enable patients and physicians to monitor symptoms in real-time and make behavioral and medication adjustments. PMDD is a chronic condition that may benefit from real-time, remote monitoring. However, clinical practice has not advanced to monitoring affective states in real-time. Identifying remote monitoring paradigms that can detect within-person affective state change may help facilitate later research on timely and efficacious interventions for individuals with PMDD. This narrative review synthesizes the current literature on behavioral and physiological correlates of PMDD suitable for remote monitoring during the menstrual cycle. The reliable measurement of heart rate variability (HRV), sleep, and physical activity, with existing wearable technology, suggests the potential of a remote monitoring paradigm in PMDD and other depressive disorders.

## 1 Introduction

**Figure 1:**
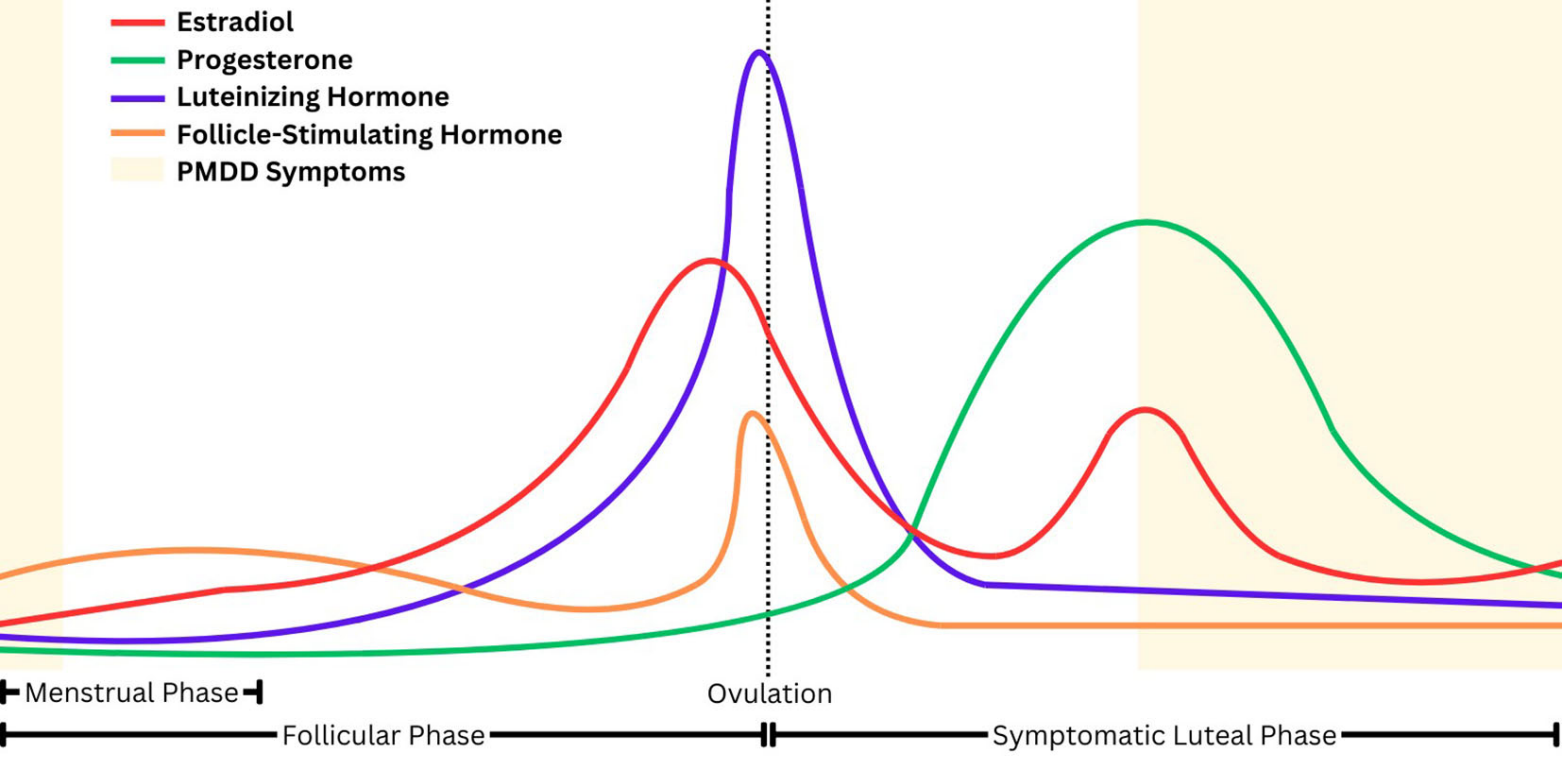
Hormones and PMDD symptom timing throughout the menstrual cycle.

Premenstrual dysphoric disorder (PMDD) is characterized by emotional, behavioral, and physical symptoms that begin in the mid-to-late luteal phase of the menstrual cycle, when estradiol and progesterone levels precipitously decline, and remit after the onset of menses (**Figure 1**) (1–3). Research suggests that the withdrawal of the neuroactive steroid allopregnanolone (ALLO), a metabolite of progesterone, during the luteal phase may diminish the effect of the inhibitory neurotransmitter gamma-aminobutyric acid (GABA) among those with PMDD, leading to a heightened stress response and reduced parasympathetic nervous system activity (3, 4).

PMDD may be thought of as a more impairing and severe manifestation of premenstrual syndrome (PMS), which is also characterized by a combination of physical and emotional symptoms that commonly include anger, depression, irritability, mood swings, breast tenderness, headache, and bloating (5). A recent meta-analysis found a 3.2% global prevalence estimate of confirmed PMDD, whereas PMS has global prevalence estimates of nearly 50% (6, 7). Like other mood disorders, the precipitous onset and remission of symptoms, which can be severe and impairing, often leaves individuals with PMDD uncertain about when symptoms will begin and the degree of impact they will have each menstrual cycle.

Management of other chronic health conditions, including cardiovascular disease and diabetes, has benefited from remote digital monitoring paradigms that enable patients and physicians to monitor symptoms in real-time and make behavioral and medication adjustments (8–12). Although digital health has not been widely adopted in mental healthcare, preliminary evidence suggests it may help individuals with depression and their healthcare providers better identify personalized patterns of risk and enable just-in-time interventions (13). PMDD is an ideal mood disorder to begin building within-person algorithms to detect mood changes, given the frequent cadence of affective switching (i.e., switching from euthymia to depression and back again each month) and the clear benefit of detecting an impending affective switch early enough to prevent or reduce its severity.

In mood disorders generally, the transition into and out of depression (i.e., “affective switching”) is characterized by increased rates of suicide (14–16). Thus, PMDD presents a unique risk due to the frequency of affective switching. Indeed, those with PMDD are seven times more likely to attempt suicide than individuals without PMDD (Prasad et al., 2021).

Despite the high mortality rate in PMDD and the associated importance of monitoring risk, PMDD is difficult to diagnose correctly and monitor over time. Specifically, both the Diagnostic and Statistical Manual of Mental Disorders, Fifth Edition (DSM-5) and the International Classification of Diseases, Eleventh Revision (ICD-11) require prospective daily assessment during two menstrual cycles (17–19). To meet the criteria for PMDD, symptoms must be present for the week before menstruation (i.e., the luteal phase), and symptoms must clear out within the first couple of days of menstruation. Per the DSM-5, at least five symptoms must be endorsed, including at least one symptom from criterion B (marked irritability or anger or increased interpersonal conflicts; markedly depressed mood, feelings of hopelessness, or self-deprecating thoughts; marked anxiety, tension, and/or feelings of being keyed up or on edge) and at least one symptoms from criterion C (decreased interested in usual activity; subjective difficulty concentrating; lethargy, easy fatigability, or marked lack of energy; marked change in appetite, overeating or specific cravings; hypersomnia or insomnia; a sense of being overwhelmed or out of control; physical symptoms such as breast tenderness or swelling, joint or muscle pain, a sensation of “bloating” or weight gain) (18).

At present, there are no commercially available apps or trackers to assist with PMDD diagnosis. The gold standard method of diagnosing PMDD calls for clinicians to use paper and pencil to hand-score prospective symptom ratings (17). Moreover, for individuals with PMDD, the timing of affective state transitions into and out of depression is contingent on the menstrual cycle. However, not all individuals have a reliably consistent cycle length, making it difficult to predict the highest-risk period (20). Ideally, clinicians would monitor those with PMDD during the highest risk period and introduce just-in-time interventions to mitigate impairment and prevent suicidality. Yet, current practice generally precludes prediction of symptom onset and timely intervention.

Other chronic conditions with heightened mortality rates have benefited from real-time remote monitoring. For example, the use of continuous glucose monitoring devices for diabetes mellitus reduces HbA1c by an additional 17-43% compared to usual care (8, 9). Similar impacts have been observed in cardiovascular disease. Compared with usual care, the use of combined remote monitoring and consultation decreases cardiovascular-related mortality and hospitalization by 17% and 28%, respectively (10). Encouragingly, 83% of adults with cardiovascular disease are willing to share wearable device data with their clinicians to improve their care (21).

Like diabetes mellitus and cardiovascular disease, PMDD is a chronic condition that may benefit from real-time, remote monitoring. However, clinical practice has not advanced to monitoring affective states in real-time (22, 23). Given that 29% of Americans already use fitness tracking devices, remote monitoring may be a feasible and affordable way to monitor affective switching (21). Consequently, using wearable devices for remote monitoring of mood disorder symptoms holds the potential for advancing population health in depressive disorders, as it has with cardiovascular disease and diabetes. However, since there is a lack of studies aimed at detecting affective switching through remote monitoring, reviewing the potential physiological biomarkers that could serve as endpoints for affective switching is warranted. PMDD, a relatively homogenous depression subtype with a known, frequent, and regularly occurring trigger of affective switching, holds promise for developing an affective state prediction model (24).

This review aims to synthesize the current literature on behavioral and physiological biomarkers of affective switching in PMDD and depressive disorders, with a focus on their suitability for remote monitoring. In particular, biomarkers were selected for review that are 1) able to be passively monitored with modern wearable technology 2) have an established association with mood and 3) have some literature supporting a relationship with the menstrual-cycle related changes. As a result, heart rate variability (HRV), sleep, and physical activity were selected for review. The review will also explore the predictive utility of passively monitoring smartphone behavior and social smartphone behavior. Finally, gaps in the existing literature will be identified and potential next steps toward applying remote digital monitoring to PMDD and other depressive disorders will be described.

## 2 Heart rate variability (HRV)

Heart rate variability (HRV), the variation in time between successive heartbeats, is a noninvasive measure of autonomic nervous system (ANS) activity, with higher HRV thought to reflect greater physiologic flexibility and ability to regulate emotional responses (25).

### 2.1 HRV measurement

HRV is measured using both time-domain measurements or frequency-domain measurements (26, 27), computed in several different ways: time-domain measurements look at the time between successive heartbeats; RR intervals refer to the time between all successive heartbeats; and NN intervals refer to the time between intervals from which artifacts, or abnormal beats, have been removed. Time-domain measures include the standard deviation of NN intervals (SDNN), the square root of the mean squared differences of NN intervals (rMSSD), and the standard deviation of the average NN intervals over a short time period (SDANN). SDNN provides an overall estimate of HRV, while rMSSD provides an overall estimate of short-term components of HRV, and SDANN provides an overall estimate of long-term components of HRV (26).

Frequency-domain measurements look at the relative power of a frequency band. Frequency domain measurements of HRV consist of very low frequency (VLF) power, low frequency (LF) power, high frequency (HF) power, and very high frequency (VHF) power. HF power represents parasympathetic nervous system (PNS) activity, while LF power can be produced by both sympathetic nervous system (SNS) and PNS activity. The LF/HF ratio is thought to represent the balance between the PNS and SNS (26, 27).

The gold standard for measuring HRV is electrocardiography (EKG), which involves measuring electric signals from the heart to measure heart activity (26, 28). HRV can also be measured with photoplethysmography (PPG) (29, 30). PPG uses LED light and a photodetector to measure the amount of light reflected by tissue, representing blood volume changes (31, 32). Thus, PPG measures pulse rate variability (PRV) as a proxy for RR intervals (29, 30). PPG devices are commonly worn on the wrist; however, newer PPG devices with increased accuracy can be worn on the finger (33).

### 2.2 Remote monitoring of HRV

Wearable devices with EKG or PPG capabilities enable remote monitoring of HRV. For example, the second-generation Oura ring, which uses PPG, demonstrated high agreement with EKG for nocturnal rMSSD (r^2^ = 0.980) (34). A systematic review of 18 studies compared HRV derived from classic EKGs with HRV derived from commercially available wearable devices (30). Results indicated correlation ranges of r=0.98-0.99 and r=0.85-0.94 for time-domain and frequency-domain indices of HRV respectively, when measured in a resting state. However, the correlations decreased when HRV was not measured in a resting state (30), indicating that wearable devices may be less accurate during activities. A separate review investigating the accuracy of PRV concluded a strong agreement between PRV and EKG when HRV is measured at rest. At the same time, the review noted that physical activity and mental stress may impair agreement. However, quantitative conclusions were precluded by heterogeneity across reviewed studies (29). Thus, currently available wearable devices are as accurate as EKG for measuring HRV and PRV during rest, though they may be less accurate during physical activity.

### 2.3 HRV and psychopathology

HRV is related to stress, including perceived stress, response to stressful life events, and adaptability to stress (35, 36). HRV has been shown to be significantly reduced in patients with major depressive disorder (MDD) and other psychiatric conditions, including schizophrenia, posttraumatic stress disorder, and bipolar disorder (37, 38). For example, two meta-analyses have demonstrated that individuals with depression have lower HRV, and lower HRV is associated with more severe depression symptoms (36, 39). However, emerging literature suggests that this association may not hold across all populations. Specifically, one study demonstrated that higher resting HRV was associated with more severe depression among Black Americans, especially among Black Americans who endorse the use of culturally compelled coping (40, 41). Thus, additional studies that investigate HRV functioning among diverse populations are needed.

### 2.4 HRV in PMDD and during the menstrual cycle

Evidence suggests that HRV changes throughout the menstrual cycle (42–45). However, this relationship may be particularly apparent for those with PMS/PMDD (46, 47). Obtaining a clear understanding of the interaction between PMDD, menstrual phase, and HRV presents challenges because 1) there are two domains of measuring HRV (i.e., frequency and time); 2) there are multiple components of HRV that can be analyzed within each domain (i.e., HF, LF, SSDN, RR intervals, SDAAN, etc.); and 3) there are multiple time points that can be compared (i.e., comparing HRV in a particular cycle phase versus comparing changes in HRV between cycle phases). Several studies have examined HRV in PMS and PMDD, both of which will be discussed to identify themes to guide future studies. Details regarding study samples, design, measurement devices, and findings of studies investigating HRV in PMS and PMDD can be found in **Table 1**.

**Table 1:** Characteristics of cited studies on HRV.

Author	Participants, n		Study Design	HRV Components	
	PMS/PMDD	Control		Time	Frequency
Baker et al. (2008) (47)	14; severe PMS (n=4) or PMDD (n=5)	15	EKG during overnight in-lab sleep study during follicular phase (6-12 days after menstrual bleeding) and late-luteal phase (9-10 days after luteinizing hormone surge)	NN^abe^, SDNN^e^, rMSSD, SDAAN	TP, HF^e^, HFpf^a^, LF/HF
Danel et al. (2019) (54)	177; PMS n=113 no hormonal contraceptive; n=64 on hormonal contraceptive		EKG continuously recorded for 10 minutes in follicular phase (4-8 days after menstruation).	SDNN^c^, rMSSD^c^	–
de Zambotti et al. (2013) (55)	12; PMS	14	EKG during overnight in-lab sleep study during follicular phase (6-10 days after onset of menstruation), mid-luteal phase (5-9 days after luteinizing hormone surge), and late-luteal phase (10-14 days after luteinizing hormone surge)	–	TP, HFpf, LF/HF
Feula et al. (2022) (51)	20; PMS	20	EKG continuously recorded for five minutes during late-luteal phase (5 to 7 days before menstruation).	SDNN^b^, rMSSD^b^	TP, HF^b^, L, LF/HF^b^
Grrishma et al. (2015) (56)	60; mild PMS		EKG continuously recorded for 5 minutes during follicular phase (8-10 days after menstrual bleeding), late-luteal phase (1-5 days before menstruation)	SDNN, rMSSD^d^	TP^d^, HF^d^, LF^d^, VLF^d^
Hamidovic et al. (2023) (50)	17; PMDD	18	Heart rate monitor during trier social stress test (TSST) during mid-luteal or late-luteal phase. HRV time points (baseline, instruction, TSST, recovery) were compared for PMS and control group	–	HF^b^
Landen et al. (2004) (52)	28; PMDD	11	EKG in both follicular phase (6-10 days after menstrual bleeding) and luteal phase (5 to 0 days before menstruation). Frequency domain HRV measured with EKG in lab for 10 minutes in standing-up position and 10 minutes in supine position. Time domain HRV measured with 24-h take-home EKG beginning on same day as frequency HRV EKG	RR, SDNN^c^, rMSSD^c^, SDANN	Supine HF^c^, standing HF, supine HF/LF, standing HF/LF
Matsumoto et al. (2007) (46)	34; PMS (n=23) or PMDD (n=11)	28	EKG continuously recorded for five minutes during mid-follicular phase (5 days after menstrual bleeding) and late-luteal phase (within 7 days before menstruation)	RR^ae^	TP^ab^, HF^ae^, LF^a^
Meng et al. (2022) (49)	50; PMS	46	EKG recorded during stress test during late-luteal phase (not defined) and follicular phase (not defined). HRV time points (baseline, task, recovery) were compared for PMS and control group	–	HF^b^, LF, LF/HF^b^
Swami & Kumar (2023) (48)	20; PMS	20	EKG continuously recorded for 15 minute during follicular phase (5 days after menstrual bleeding), late-luteal phase (7 days before menstruation)	RR^a^, SDNN^b^	HF^d^, LF, VHF^ad^, VLF^ad^, LF/HF^ad^

RR, regular intervals; NN, normal to normal intervals; SDNN, standard deviation of NN intervals; rMSSD, root of the mean square differences of NN intervals; SDANN, standard deviation of the average NN intervals over a short period of time; TP, total power; HF, high frequency power; HFpf, high frequency peak frequency; LF/HF, ratio of low frequency and high frequency power; LF, low frequency power; VHF, very high frequency power; VLF, very low frequency power; ^a^, PMS/PMDD and control group significantly different overall; ^b^, PMS/PMDD and control group significantly different within luteal phase; ^c^, PMS/PMDD and control group significantly different within follicular phase; ^d^, PMS/PMDD group significantly different in luteal phase compared to follicular phase, not considering control group; ^e^, PMS/PMDD significantly different in luteal phase compared to follicular phase for PMS/PMDD group, but not control group.

#### 2.4.1 Differences in HRV between PMDD, PMS, and asymptomatic control groups during the menstrual cycle

Individuals with PMS/PMDD have been shown to differ from those without PMS/PMDD in certain HRV metrics, regardless of cycle phase. While some studies indicate that those with PMS/PMDD experience lower HRV, on average, results are mixed across studies and thus may be driven by specific HRV components. For instance, while Matsumoto et al. (2007) found that individuals with PMDD have lower HF power throughout the menstrual cycle compared with individuals with PMS or no premenstrual symptoms, Baker et al. (2008) and Swami and Kumar (2023) found that overall HF power was not significantly different between groups (46–48). In contrast, Swami and Kumar found lower VHF power in those with PMS compared to controls throughout the menstrual cycle, and Baker et al. found smaller mean NN intervals in those with PMS compared with controls throughout the menstrual cycle (47, 48).

Results are similarly mixed with regard to LF power. Matsumoto et al. found that LF power was lower in those with PMDD compared with those with PMS and those without PMS/PMDD; Swami and Kumar did not find any group differences; and Baker et al. did not report LF power (46–48). A similar pattern emerged with the LF/HF ratio, where Swami and Kumar found an increased ratio in those with PMS overall; Baker et al. did not find any group differences; and Matsumoto et al. did not report the LF/HF ratio (46–48). Finally, Matsumoto et al. found decreased total power in the PMDD group compared with the PMS and control groups; while Baker et al. did not find any group differences; and Swami and Kumar did not report total power (46–48). Thus, the literature is mixed with regard to differences between PMDD, PMS, and control groups in HRV and its components when measured during the menstrual cycle rather than examining specific cycle phases.

#### 2.4.2 Differences in HRV between PMS and asymptomatic control groups within specific cycle phases

Some studies have examined components of HRV between those with PMS and asymptomatic controls during the follicular phase, luteal phase, or both. For instance, two studies that implemented a social stress test in the luteal phase indicated a delay in HF power recovery after the stress task for the PMS group, compared with controls (49, 50). An additional study indicated lower SDNN and rMSSD in the luteal phase in those with PMS compared with controls (51). However, one study found a lower SDNN, rMSSD, and HF power in the follicular phase for the PMS group compared with controls and did not indicate any within or between-group differences within the luteal phase (52). Similarly, a study that investigated the relationship between PMS symptoms and HRV during the follicular phase found a positive association between PMS symptoms and SDNN and rMSSD for those not on hormonal contraceptives, which is the opposite direction of the effect one would expect based on Landén et al., 2004 (52). This effect did not remain among those on hormonal contraceptives, which could be because hormonal contraceptives have been shown to stabilize the hormonal shifts that occur during the menstrual cycle and reduce PMS symptoms (53, 54). Taken together, these studies suggest HRV and its components are lower in the symptomatic luteal phase in those with PMS compared with those without PMS.

#### 2.4.3 Within-group changes in HRV during the menstrual cycle in PMDD

A considerable number of studies have indicated that the symptomatic luteal phase is characterized by reduced HRV in those with PMS/PMDD. Specifically, women with PMS/PMDD show decreased HF power in the luteal phase compared with the follicular phase (46–48, 55, 56). Additionally, individuals without PMS/PMDD do not show cycle phase differences in HF power, suggesting the luteal phase reduction in HRV is unique to those with PMS/PMDD (46, 47, 55). Time-domain measures such as SDNN and rMSSD are also lower in the luteal phase compared to the follicular phase for those with PMS/PMDD (47, 48, 56). Thus, studies have consistently shown certain aspects of HRV are lower in the luteal phase compared with the follicular phase in those with PMS/PMDD, but not in asymptomatic controls.

#### 2.4.4 Methodological challenges and HRV summary

Across the three sets of studies reviewed, methodologic variation may account for important differences in findings. Of the studies reviewed, two measured HRV during sleep (47, 55), two measured HRV during a stress test (49, 50), and the remaining six measured HRV with a supine or standing EKG sample of varying lengths of time (46, 48, 51, 52, 54, 56). Additionally, as indicated in **Table 1**, different components of HRV are reported in each study, precluding a full comparison of study results. Finally, sample sizes are consistently small or moderate, and small sample sizes may obscure true findings and may also contribute to false discoveries. Adequately powered studies are needed to determine the extent to which HRV may be associated with the onset of mood symptoms, physiologic symptoms, or their combination during the menstrual cycle in those with PMDD, PMS, and asymptomatic controls.

Despite these methodologic differences, one clear and consistent pattern of results emerged. Across studies, some components of HRV were lower in individuals with PMS/PMDD during the luteal phase compared with the follicular phase, and this difference was unique to individuals with PMS/PMDD. This suggests that HRV variation may be a valid physiologic marker of within-person symptom variation in those with menstrually-related mood disorders (i.e., PMS or PMDD). In contrast, while some studies indicate PMDD is defined by lower HRV across the menstrual cycle compared with those with PMS and asymptomatic controls, these results have not been consistently replicated across studies and should be interpreted with caution. As such, HRV may not be a good diagnostic marker for PMDD.

## 3 Sleep

### 3.1 Sleep measurement

In the literature, sleep is typically assessed by examining the duration of sleep, sleep staging, or both using multi-modal physiologic assessment (**Table 2**). Polysomnography (PSG) uses a combination of electroencephalogram (EEG), electrooculogram, electromyogram, EKG, pulse oximetry, and airflow and respiratory effort to determine wakefulness and sleep as well as staging (57). PSG offers a comprehensive look at the structural organization of sleep, or sleep architecture, and is considered the gold standard for measuring sleep and diagnosing sleep disorders (58, 59).

**Table 2:** Components of sleep architecture (59, 167, 168).

REM sleep	Rapid eye movement sleep
NREM sleep	Non rapid eye movement sleep
Stage 1 sleep	Brief period transitioning from wake to sleep, or “dozing off” period
Stage 2 sleep	Light sleep
Stages 3 and 4 sleep	Slow wave sleep or deep sleep
Total sleep time (TST)	Time spent in REM or NREM sleep
Sleep onset latency	Time it takes to fall asleep
Sleep efficiency	Ratio of total time asleep to time spent in bed intending to sleep
Wakefulness after sleep (WASO)	Time spent awake after sleep onset but before final awakening

Alternative ways to measure sleep physiology include actigraphy and photoplethysmography (PPG). Actigraphs are wearable devices, typically worn on the wrist, that measure sleep by detecting physical movements. Most modern actigraphs include accelerometers for movement detection (60). Additionally, PPG measures heart rate, HRV, blood oxygen saturation, and respiratory rate, which can be used to indicate sleep (32, 33, 61).

Subjective sleep measures, such as sleep diaries or questionnaires, prompt an individual to retrospectively report on sleep components (e.g., time in bed, sleep onset latency). While the most common subjective sleep measures demonstrate strong internal consistency and test-retest reliability, subjective sleep measures are not strongly correlated with objective sleep measures (62–64). In particular, the accuracy of self-reported sleep quality is vulnerable to being impacted by memory processes, personality, mood states, and subjective well-being (64–66). However, subjective sleep measures are low-cost and highly feasible while offering some insight into sleep habits and may help place physiological sleep assessments into context (e.g., knowing that an individual woke up several times in one night because of a thunderstorm can help with the interpretation of physiologic measures).

### 3.2 Remote monitoring of sleep

PSG typically involves an individual spending at least one night sleeping in a sleep laboratory setting and consists of a specialist observing and interpreting the gathered PSG sleep data. Despite PSG being the gold standard of sleep measurement, wearable devices therefore offer a more unobtrusive, affordable, and feasible way to monitor sleep on an ongoing basis. In determining the validity of remote sleep monitoring devices, attention is paid to sensitivity (i.e., ability to detect sleep), specificity (i.e., ability to detect wake), and staging (i.e., ability to detect sleep stage) (67, 68).

Actigraphy can assess sleep-wake patterns in individuals with average or good sleep with reasonable reliability and validity compared to PSG (60, 69, 70). Additionally, the accuracy of consumer actigraphy devices is comparable to that of research-grade actigraphy devices (71–73). Actigraphy has strong sensitivity (ability to detect sleep) but tends to overestimate total sleep time. However, specificity (ability to detect wake) is consistently low (32, 74–77). Moreover, accuracy may diminish among people with lower sleep quality depending on the device being used (74, 78–80). Another disadvantage of actigraphy is its lack of validation for identifying sleep stages (68, 81). Taken together, these studies suggest that both actigraphy and commercial grade wearable devices can validly measure sleep initiation and duration.

Newer consumer devices include a combination of PPG, accelerometry, and body temperature to achieve increased sleep/wake scoring accuracy compared to actigraphy alone. Moreover, PPG can predict sleep staging with moderate accuracy compared to PSG (32, 68, 71, 82–84). With few exceptions, PPG-based devices that classify sleep into three or four stages have 65-75% staging accuracy (68). Despite this potential for remotely monitoring sleep staging, consumer wearable devices have distinct disadvantages in the research context: 1) the scoring algorithms used by the consumer devices are often proprietary and 2) ongoing improvements to these algorithms may impact within-person reliability during ongoing sleep studies (32).

Newer studies have begun to look at wearable and portable EEG devices, such as in-ear or headband EEG devices. Some wearable EEG devices, such as the Dreem headband or an in-ear EEG, may be more accurate than accelerometers and PPG and are capable of identifying all five sleep stages when used properly (32, 68, 82, 85, 86). Thus, portable and wearable EEG technologies hold promise for studying sleep/wake and sleep staging.

### 3.3 Sleep and psychopathology

Sleep disturbances are transdiagnostic precipitants and symptoms of MDD and other psychiatric disorders, including bipolar disorder and schizophrenia (87–89). Sleep disturbances are thought to indicate an underlying circadian dysfunction in MDD and mood disorders more generally (90), though circadian dysfunction has not been well studied in PMDD.

### 3.4 Sleep and the menstrual cycle

Sleep varies by menstrual phase among menstruating individuals, irrespective of PMS/PMDD status. The exact nature of this relationship, however, is not fully understood. Women generally report decreases in perceived sleep quality in the luteal phase compared with the follicular phase (91, 92). One study of 163 women used actigraphy to measure sleep and found that sleep efficiency declined gradually across the menstrual cycle, with a more apparent decline in the luteal phase. However, participants were not all regularly menstruating and were also in different stages of the reproductive life cycle (pre-, early-, and late-perimenopause) (92). Thus, the relationship between sleep efficiency and the menstrual cycle may be somewhat obscured by the inclusion of those who were not regularly menstruating.

Additional studies have directly examined the relationship between sleep and ovarian hormone changes during the menstrual cycle. Rising progesterone levels have been associated with objective sleep measures, including decreased sleep HRV (55) and increased PSG-measured sleep disturbances (93). A recent review found that endogenous progesterone has a sleep-promoting effect and that hormone-related sleep problems were more associated with the rate of change in reproductive hormones than the absolute levels of hormones (94). Taken together, progesterone may regulate sleep during the menstrual cycle in regularly menstruating individuals and may be responsible for cycle phase effects.

### 3.5 Sleep and PMDD

The menstrual cycle may have a greater impact on sleep among those with PMS or PMDD who report higher levels of insomnia and fatigue and perceive lower sleep quality throughout the entire menstrual cycle compared to those without PMS/PMDD, with the greatest differences occurring during the luteal phase (47, 92, 95–98). However, few studies have examined objective measures of sleep in this population (99).

Baker et al. (2012) compared objective and subjective sleep measures in 18 women with severe PMS and 18 women with minimal menstrual symptoms. The PMS group exhibited poorer subjective sleep quality in the luteal phase and increased levels of slow-wave sleep, as measured by PSG, throughout the menstrual cycle, compared with controls (100). Similar results were found by Shechter et al. (2012), where women with PMDD and luteal-phase insomnia (n=7) experienced more slow-wave sleep during the luteal phase compared with a control group (n=5). However, the sample size was small and the control selection criteria were not well defined (101). In a study done by de Zambotti et al. (2013) the PMS group (n=12) appeared to spend more time in slow-wave sleep in the luteal phase compared with controls (n=14) (17.4 vs 14.3% TST in mid-luteal; 16.2 vs 11.3% TST late-luteal), yet, these results did not reach significance (55).

In contrast, earlier studies indicated that individuals with PMS/PMDD display decreased slow-wave sleep compared to a control group during both the follicular and luteal phases although small sample sizes limit these findings (84, 95). A larger study (n=23 PMDD; n=18 controls) found no difference in slow-wave sleep during the mid-follicular phase or the late-luteal phase, however, results should be interpreted within the context of a clinical trial looking at sleep deprivation therapy (102).

The relevance of sleep in PMDD is further indicated by a series of studies that demonstrated a delayed reduction in endogenous melatonin levels in mornings during the luteal phase compared with the follicular phase in those with PMDD (102–106).

Overall, future studies should focus on delineating the relationship between PMDD and sleep at the within-person level to determine if remote sleep monitoring devices can be used to predict or detect affective switching.

## 4 Physical activity

### 4.1 Physical activity measurement

Physical activity can be measured via self-report, accelerometers, pedometers, heart rate monitors, and sensors that combine different measurement modalities (108, 109). Aspects of physical activity that can be measured may include energy expenditure, step count, distance traveled, and time spent in different postures. The gold-standard method for measuring physical activity involves quantifying energy expenditure using the doubly labeled water method, which entails measuring elimination rates of specific isotypes following the ingestion of deuterium and heavy oxygen-labeled water (110). This method is expensive, burdensome, and time-intensive and is therefore not feasible for remote monitoring of physical activity (for a review, see Sylvia et al. (108).

### 4.2 Remote monitoring of physical activity

Wearable devices are overall an accurate and feasible way to track physical activity, although validity varies between brand. Fuller et al. (2020) conducted a systematic review of commercially available wearable devices for measuring steps, energy expenditure, and heart rate. The review indicated that criterion validity depends on the device, study type (controlled or naturalistic), and type of measurement. Validity for step count was best for Apple Watch and Garmin, while Fitbit, Samsung, and Withings were within +/-3 mean percentage error on average. Heart rate was also accurately measured; all brands fell within +/-3 mean percentage error on average, with a small tendency for underestimation. Wearable devices were found to be unreliable for measuring energy expenditure (111). However, this review did not include devices designed to be worn on the finger (e.g., Oura ring), which emerging studies demonstrate to be highly correlated with gold-standard measures of step counts, heart rate, and energy expenditure (112, 113).

### 4.3 Physical activity and psychopathology

Depressive symptoms and physical activity have a well-established link. A meta-analysis of 42 studies reported a significant inverse relationship between physical activity (i.e. actigraphy or pedometer) and rates of depression. However, these findings were based on cross-sectional studies, so the directionality of the effects cannot be inferred (114). Decreased physical activity has been consistently linked to risk for depression, although findings regarding the impact of depression on subsequent physical activity are mixed (115–119) Nevertheless, objective measures support a strong negative relationship between depressive symptom severity and daily step count (118, 119).

A wealth of research has been conducted on the effectiveness of physical activity as an intervention for depression. Hu et al. (2020) conducted a systematic review of eight meta-analyses across 134 studies concerning exercise as an intervention for depression symptoms. They concluded that exercise interventions have a moderate effect on reducing depressive symptoms (120). A separate systematic review of 13 studies reported that 10 studies showed a statistically significant reduction in depression symptoms following a randomized-controlled exercise intervention. The review concluded that any physical activity for 30-45 minutes at least three times a week, preferably performed under supervision, is recommended to treat MDD (121). Given both the naturalistic and experimental results linking depression and physical activity, physical activity may be a reliable physiologic indicator of depressed mood.

### 4.4 Physical activity, the menstrual cycle, and PMDD

To date, there is a lack of research on the relationship between physical activity, the menstrual cycle, and PMDD. A recent meta-analysis on the effects of the menstrual cycle phase on exercise indicated that there may be a trivial reduction in exercise during the early follicular phase (122). A separate study indicated no reduction in step count as a result of menstrual phase (123). Another review looked at the performance of athletes throughout the menstrual cycle and concluded mixed findings regarding levels of physical activity or athletic performance and the menstrual phase (124).

Studies investigating changes in physical activity throughout the menstrual cycle among individuals with PMS/PMDD are lacking. However, one study indicated that women with severe PMS walked 1,411 fewer steps during the luteal phase and menses compared with asymptomatic control women (125). Additionally, observational studies support a negative relationship between looking at PMS/PMDD symptoms and general exercise (126–128). Additionally, there is growing evidence supporting physical activity as an effective intervention for PMS (128, 129). A systematic review of five RCTs with 492 participants concluded that aerobic exercises effectively improve premenstrual symptoms (130).

Physical activity and depression symptom severity are likely bidirectionally related. As depression is a common feature of PMDD, more research is warranted to determine the extent to which objective measures of physical activity can be used to predict PMDD symptoms.

## 5 Social behaviors and smartphone use

### 5.1 Remote monitoring of social behavior with smartphones

Aspects of social behavior can be gleaned by tracking smartphone use. For instance, smartphone use for interpersonal connection is positively associated with a higher likelihood of participating in social activities (131), greater belonging support, and greater tangible social support over time. Problematic smartphone use, involving an excessive psychological attachment to one’s smartphone, is associated with less tangible social support over time (132). Thus, smartphone activity, and specific types of smartphone activity, may be a feasible proxy for social behavior.

### 5.2 Remote monitoring of mood with smartphones

Smartphone data may be associated with mood. Objectively monitored speech patterns from smartphone voice data can predict mood states with up to 97.4% accuracy (133, 134). Applying machine learning models to passively collected smartphone data has been shown to accurately detect fluctuations in mood states, including in those with MDD (135–138). Given the established predictive utility of passively collected smartphone-use data on mood fluctuations, applying these findings to affective switching among people with PMDD is a promising area for future investigation.

### 5.3 Social media use and mood

Research strongly supports a relationship between social impairments and depressive symptoms (139–141). Moreover, social interaction and support are known to influence clinical outcomes in depression (142–144). The emergence of smartphones and social media introduces new considerations when studying social impairment. While some social media interactions improve mood, most studies show that increased time spent engaging with cell phones and social media apps is associated with greater depression severity (145–149).

Studies of social media use and mood have produced mixed findings. A systematic review of 13 studies investigating adolescent social media use demonstrated a positive association between psychological distress and social media use across multiple measures (150). However, a separate review noted that research on social media use and adolescents has been mostly cross-sectional and has generated conflicting results and small effect sizes (151). Another review indicated a *positive* association between social media use and mood (152). Social media findings are thus hard to interpret. More detailed studies measuring how an individual uses social media will likely provide better information about the impact of social media on mood. So far, studies of the type of social medial interactions (i.e., active vs passive, private vs public) have yielded similarly mixed findings (153–155).

### 5.4 Social impairment, PMDD, and the menstrual cycle

PMDD is associated with social impairment during the luteal phase, including interference in relationships with friends, classmates, and coworkers (156–160). Rubinow and colleagues (2017) administered a Facial Discrimination Task in the luteal and follicular phases of women with PMDD and asymptomatic controls. They found that women with PMDD exhibited increased negative judgments and impaired specificity of judgments during the luteal phase compared with the follicular phase, while controls did not experience any menstrual effects (157). These findings suggest that facial recognition is impaired during the luteal phase in PMDD, which could have downstream effects on social behavior.

Women with PMDD also report higher levels of hostility regardless of menstrual phase (158) and more aggressive tactics to solve conflict during the luteal phase (161). Kaiser and colleagues found that among women with PMDD, pain and somatic dysphoria in the luteal phase is correlated with impairment in social activities, while premenstrual irritability in the luteal phase is correlated with impairment in relationships (159). These findings support previous research suggesting that those with PMDD suffer from increased irritability in the luteal phase, which could negatively impact social engagement (158, 162).

Overall, considering the feasibility of smartphone data collection, future research regarding the predictive utility of smartphone data both generally and as a proxy for social behavior among those with PMDD is warranted. However, current research methods of social media use are crude proxies for more nuanced social interactions that could be collected by monitoring social media and smartphone use.

## 6 Discussion

This review synthesized the current literature on behavioral and physiological correlates of PMDD suitable for remote monitoring during the menstrual cycle. PMDD is marked by the onset and offset of a depressive state provoked by hormonal fluctuations during the menstrual cycle. Switching into and out of depressive states is associated with an increased risk of suicide, therefore, periods of affective switching may be important to monitor to enable just-in-time interventions. Given the cyclical and chronic nature of affective switching in PMDD and attendant suicide risk, identifying remote monitoring paradigms that can detect within-person affective state change may help facilitate later research on timely and efficacious interventions. The reliable measurement of key physiologic variables associated with depression symptoms, HRV, sleep, and physical activity, with existing wearable technology, suggests the potential of a remote monitoring paradigm in PMDD.

### 6.1 HRV

HRV is an indicator of ANS activity that can be effectively monitored with remote wearable devices, particularly during rest period (29, 30, 34). HRV has been found to relate to stress and depression severity, and is significantly reduced in patients with mental illness (35–39). Although few studies have examined the relationship between PMDD and HRV, recent evidence suggests reduced HRV during the symptomatic luteal phase in those with PMS/PMDD (46–48, 55, 56). Findings consistently demonstrate decreased HF power during the luteal phase, which can be interpreted as a reduction in overall PNS activity (26). These findings align with MDD literature that indicates lower HRV in individuals with symptomatic MDD, compared with controls (36, 39). Although existing studies demonstrated group-level reductions in HRV between the follicular and luteal phases in those with PMDD/PMS, additional research to establish within-person changes in HRV or HF power will be needed to establish the use of this variable as a correlate or predictor of symptom onset to guide clinical practice.

### 6.2 Sleep

Sleep disturbances are an established precipitant and symptom of psychiatric disorders that can be tracked easily and accurately with remote monitoring (68, 87, 88, 90). In particular, remote monitoring is an effective tool for capturing total sleep-wake time, and newer technology has begun to track sleep staging reliably (32, 68, 71, 82–86). Individuals with PMS/PMDD have more of a negative perception of sleep quality, particularly heightened during the luteal phase, compared to those without PMS/PMDD (47, 92, 95–98). Evidence suggests that the circadian rhythm may be disturbed in the luteal phase among those with PMS/PMDD, with some indications of altered melatonin secretion and slow-wave sleep (55, 100–107). However, because some studies indicate that sleep abnormalities persist throughout the menstrual cycle without showing phasic differences, sleep may be a less useful metric of affective switching in PMDD. Despite this, the prominent finding that perceived sleep quality diminishes in the luteal phase should not be disregarded. It is plausible that sleep quality perception is influenced by psychological state rather than actual sleep quality. However, it is also plausible that changes in perceived sleep quality can be attributed to changes in sleep architecture that are not detectable with between-person study designs. Future studies should focus on delineating the relationship between PMDD and sleep at the within-person level to determine if remote sleep monitoring devices can predict affective switching and help inform the implementation of effective sleep interventions.

### 6.3 Physical activity

Physical activity can be easily and accurately tracked with remote monitoring methods (111–113). Despite a well-established relationship between depression and physical activity, the bidirectional nature of this relationship has not been well articulated (114–119). In PMS/PMDD, there is not enough evidence that physical activity and exercise (both performance and amount) vary by menstrual phase. Research regarding physical activity/exercise as an intervention is more well-established. Evidence suggests that exercise can meaningfully reduce depression symptoms among those with a depressive disorder (120, 121). Further, growing evidence supports physical activity as an effective intervention for PMS/PMDD (128–130). Additional research is needed to determine whether objective measures of physical activity can be used to predict PMDD symptom onset.

### 6.4 Social behaviors and smartphone use

Machine learning models applied to passively collected smartphone data to predict mood and social behavior (133–138). Thus, the justification for studying PMDD and smartphone data is two-fold. First, PMDD is marked by social impairment during the symptomatic luteal phase (156, 157, 159, 160, 162). Thus, passively collected smartphone data may be a feasible and unobtrusive proxy for social behavior that can identify affective switching and inform effective social interventions. Second, smartphone data has been demonstrated to predict affective switching with reliable accuracy among individuals with psychiatric illnesses (133–138). Thus, studying the application of machine learning capabilities to model smartphone use in individuals with PMDD is a logical next step. Overall, smartphone data has the potential to reliably predict affective switching among those with PMDD and be used as a marker of social behavior. However, more granular data regarding social communication with smartphones and social media seem necessary, compared with rough metrics of smartphone and social media use.

### 6.5 Theoretical model of affective switching in PMDD

Based on prior research, withdrawal of the neuroactive steroid allopregnanolone (ALLO) during the luteal phase may diminish the inhibitory effect of the inhibitory neurotransmitter gamma-aminobutyric acid (GABA) among those with PMDD, leading to a heightened stress response and reduced parasympathetic nervous system activity (3, 4). Although not fully understood, in mammals, GABA activity may modulate the activity of neurons in the suprachiasmatic nucleus, which regulates melatonin secretion (163, 164). Thus, alterations in GABA functioning might have downstream effects on melatonin secretion and circadian rhythms, contributing to sleep disturbances. As a result, emotional regulation may become compromised by reduced GABA function and fatigue. Although the exact mechanisms of HRV are unclear, both depression symptoms and GABAergic activity may lead to decreased HRV, further impairing stress and emotion regulation abilities. The subsequent cycle of stress, fatigue, and depressive symptoms may yield social withdrawal and inactivity, creating a compounding effect on overall well-being. Importantly, the endpoints of sleep disturbances, HRV, physical activity, and social engagement can be unobtrusively monitored with widely used wearable devices and smartphones.

### 6.6 Limitations

Given that PMDD was not added to the DSM until 2013, there is less research on PMDD as a diagnostic entity, and earlier studies included those with PMDD in studies of PMS. Thus, the extent to which PMDD and PMS are distinct or overlapping entities concerning physiologic markers remains somewhat unclear. The research conducted since 2013 seems to indicate that PMDD is distinct from asymptomatic controls with regard to certain physiologic markers, while those with PMS appear similar to those with PMDD in some studies and more similar to controls in others. Due to the relative lack of research focusing specifically on PMDD, PMS studies were included. However, PMS findings should be considered preliminary as they pertain to individuals with PMDD.

Moreover, existing studies on PMS or PMDD often included small sample sizes and had certain methodological issues. For example, PMDD diagnoses in the reviewed studies were not always based on the gold standard prospective reporting method. Additionally, existing studies did not always control for factors that may affect mood states and the menstrual cycle. For example, study results can be impacted by hormonal contraceptive use, comorbid diagnoses, cycle regularity, pregnancy status, and demographic factors such as age, race, or ethnicity (165). Although assessing and controlling for these factors can be challenging, future studies should measure and report such variables, and where appropriate, control for these confounding variables in statistical analyses.

Additionally, existing studies have predominately been conducted on cis-gendered women and neglect to consider the impact of alternate gender identities (transgender, non-binary, gender non-conforming, etc.). As such, those who do not identify as a cis-gendered woman are underrepresented in this area of research. Because people of alternate gender identities are at heightened risk for adverse mental health outcomes, excluding this population may perpetuate systemic barriers to accessing care (166). Considering gender identity in analyses, oversampling non-cisgender individuals, or not excluding people of non-cisgender identities is imperative.

## 7 Conclusion

PMDD is marked by frequent affective switching, with depressive symptoms beginning during the luteal phase and ending shortly after the onset of menses. Affective switching is a period of increased risk of suicide. Given the frequency of affective switching and the chronicity of PMDD, identifying an unobtrusive strategy for identifying periods of heightened risk could enable the delivery of just-in-time interventions. Additionally, given the frequency of affective switching, PMDD may serve as an ideal model for prospectively identifying physiologic markers of affective switching that could be applied to identifying depressive episodes in other depressive disorders that are more difficult to predict.

Remote monitoring is a promising, non-invasive, and passive mechanism for predicting affective switching and providing real-time intervention, as exemplified in chronic conditions such as diabetes and heart conditions. Prospectively identifying within-person physiological and behavioral correlates and predictors of affective switching suitable for remote monitoring is the first step in implementing such a strategy for PMDD. Whether phase-dependent variations in HRV, sleep, physical activity, social variations, and smartphone data that can be monitored remotely will be able to predict affective switching at the individual level will require additional research. If these physiologic variables predict within-person affective switching in those with PMDD, remote monitoring would hold tremendous promise for advancing population health by identifying personalized, scalable intervention strategies for those with PMDD.
